# Characterization of Volatilized Compounds in Conventional and Organic Vegetable-Source Alternative Meat-Curing Ingredients

**DOI:** 10.3390/molecules30040835

**Published:** 2025-02-11

**Authors:** Siyuan Sheng, Erin M. Silva, Steven C. Ricke, James R. Claus

**Affiliations:** 1 Meat Science and Animal Biologics Discovery Program, Department of Animal & Dairy Sciences, University of Wisconsin-Madison, Madison, WI 53706, USA; sricke@wisc.edu; 2 Department of Plant Pathology, University of Wisconsin-Madison, Madison, WI 53706, USA; emsilva@wisc.edu

**Keywords:** GC-MS/MS, steam distillation, alternative meat curing, celery, Swiss chard, volatile compounds

## Abstract

This study investigates the volatile compounds that contribute to the unique flavor and aroma profiles of cured meat products using alternative ingredients, specifically focusing on commercially available, conventional, and organically produced pre-converted celery (*Apium graveolens*) and Swiss chard (*Beta vulgaris* subsp. maritima) juices and powders. Volatile compounds were isolated and analyzed using an optimized method involving steam distillation with liquid–liquid phase extraction coupled with gas chromatography–tandem mass spectrometry (GC-MS/MS). The key volatile compound identified in celery was 3-butylisobenzofuran-1(3H)-one, and in Swiss chard, 2-methoxy-4-vinylphenol. In both conventional and organic celery juice, senkyunolide, sedanolide, and limonene were the primary volatiles, listed in descending order of concentration. This pioneering work on volatile and aromatic compounds in alternative curing ingredients provides foundational knowledge for sensory and volatile compound studies in alternative meat curing. It also offers valuable insights for organic plant and meat producers, processors, and consumers. Practically, this research highlights volatile chemicals that could interact with other meat constituents or residues in finished products, informing and enlightening future studies on the sensory and aromatic properties of alternative cured meats. Overall, this study contributes to the development of alternative cured meats, supporting the research and innovation of organic meats.

## 1. Introduction

In the curing of processed meat products, salt, nitrite, or nitrate, are formulated into fresh meat ingredients, resulting in the creation of desirable valued-added products with unique sensory attributes [1]. Nitrite and nitrates are traditional meat-curing ingredients and have been well-documented for their efficacy in preserving meat from lipid oxidation [2], providing characteristic cured colors and flavors [3], and preventing the growth of pathogenic bacteria, including Clostridium botulinum and other harmful microorganisms [4]. However, purified forms of nitrite used in traditional processing are not permitted by the USDA National Organic Program (NOP). Consequently, organic meat processors are required to use alternative curing ingredients [5].

Celery (*Apium graveolens*) powder is the most common alternative curing ingredient used in the United States [6]. Celery powder contains naturally occurring nitrates that can be converted to nitrites during the curing process. It can also be added to processed meats in a pre-converted form. Swiss chard powder (*Beta vulgaris* var. cicla) is another commonly available meat-curing powder. It contains similar or higher levels of nitrate compared to celery powder, making it an effective alternative [7]. Moreover, it generates an unnoticeable off-flavor, making it more favorable for consumers with celery allergies [8]. In addition, cherry or acerola powder was found to be a good candidate to replace sodium erythorbate as a curing accelerator.

The U.S. Department of Agriculture (USDA) regulates meat products that use natural curing agents, requiring them to be labelled as “Uncured” or “No Nitrate or Nitrite Added…” [9]. In alignment with the clean label trend and consumer awareness of organic foods, the organic meat market is experiencing significant growth, driven by increasing health consciousness and concerns regarding overuse of additives and chemicals [10]. The market size is projected to grow from USD 20.27 billion in 2024 to USD 29.71 billion by 2029, with a compound annual growth rate (CAGR) of 7.94% [11]. The National Organic Standards Board (NOSB) currently permits the use of non-organic celery powder in organic products. However, they are in the process of conducting sunset reviews, aiming to transition to organic sources of celery powder. This involves evaluating whether non-organic celery powder should remain on the National List of Allowed and Prohibited Substances or be replaced with organic alternatives [12]. Comparative studies have shown that nitrate content in conventionally grown vegetables, using mineral fertilization, was higher than those grown organically [13]. Moreover, organic fertilizers significantly increased the production of total phenolics, flavonoids, ascorbic acid, saponins, and glutathione in the vegetables compared to inorganic fertilizers [14]. Studies have shown that volatile profiles of vegetables varied significantly between conventional and organic growing conditions [15,16,17]. A more recent study has shown that celery cultivars exhibited unique volatile profiles, with significant differences observed among the cultivars [18]. It remains unclear whether organic and conventionally produced pre-converted celery and Swiss chard curing ingredients have similar chemical and volatile profiles. Additionally, the impact of organic fertilizer application on the volatile and aromatic compounds in these vegetables is not well understood.

Current research has focused on sensory attributes of processed alternative cured meats other than the volatile compounds profile in alternative curing ingredients that could influence processed meats’ sensory attributes. There is a significant gap in understanding the profiles of volatile and aromatic compounds in pre-converted alternative curing powders and juices. Understanding the volatile compound profile of alternative ingredients can provide valuable insights into how they contribute to the flavor and aroma of cured meat products. This knowledge is essential for meat processors and product developers, as it enables them to formulate products with favorable sensory attributes. Understanding volatile compounds facilitates the comprehension that alternative curing methods can yield cured meats with consistent and appealing flavor profiles. Thus, this study aims to addresses this knowledge gap by characterizing the profiles of aromatics using a GC-MS/MS system with steam distillation on commercially available conventional and organic fresh celery juice, pre-converted organic celery juice, and commercially available conventional and organic celery and Swiss chard powders. This research establishes a foundation for future studies on sensory attributes and volatile compounds in alternative meat-curing processes. Ultimately, it contributes to the development of meat products that cater to consumer demand for natural, safe, and flavorful options.

## 2. Results

### 2.1. Physicochemical Properties of Commercially Available Pre-Converted Vegetable Curing Powders

As shown in Table 1, this study assessed four pre-converted conventional and organic celery- and Swiss chard-based vegetable meat-curing powders currently available on the United States market. Pre-converted conventional celery, Swiss chard, and organic Swiss chard were supplied by Florida Foods Inc. (Eustis, FL, USA), and the pre-converted celery powder was supplied by Diana Foods Inc. (Antrain, France). From the ingredient specification (Table 1), the commercially available vegetable powders exhibited color appearances ranging from yellowish to brownish, with aroma attributes typically characterized by a nutty, woody, or vegetable-like smells.

The declared value from ingredient certificate of analysis on those pre-converted vegetable powders ranged from 20,250 to 24,750 ppm with a median value of 22,500 ppm sodium nitrite equivalent (Table 1). The nitrite concentration by an HPLC system (ENO-20 NOx Analyzer, Eicom Inc., Kyoto, Japan) in those powders ranged from 17,262 to 22,487 ppm sodium nitrite equivalent. The pre-converted vegetable powders were preserved in a sealed bag with silicon dioxide as anti-caking agent and the pH value ranged from 7.49 to 8.89. The pH condition of the pre-converted vegetable powder was within an ideal range for preserving nitrite content, preventing it from dissipating and oxidizing to nitrate [19].

Subsequent analyses were conducted on the volatile compounds present in commonly available conventional and organic curing powders to further understand their potential effects on meat curing. Additionally, an analysis of volatile compounds in conventional and organic curing accelerators (cherry powder) was performed (total ionizable compounds graph is depicted in Figure S3). Results showed that the accelerators contained negligible amounts of volatile compounds (Table 2), with no significant differences (*p* > 0.05) observed among the powders (Table 3).

### 2.2. Comparison of Commercially Available Conventional and Organic Pre-Converted Vegetable Curing Powders

**Figure 1:**
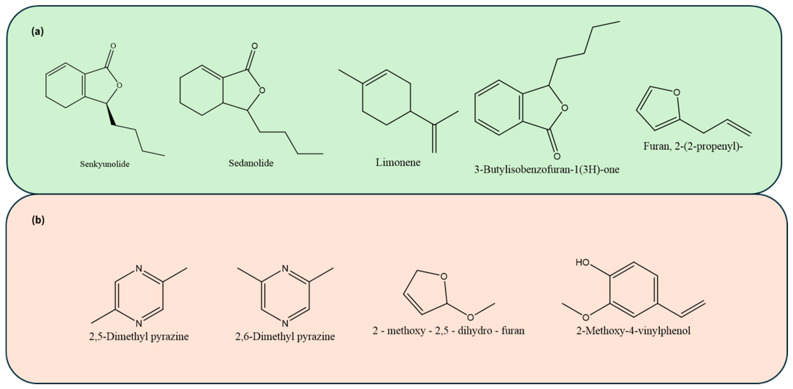
Chemical structures of key volatile compounds identified in (**a**) alternative vegetable curing juice and (**b**) commercially available alternative curing powder.

**Figure 2:**
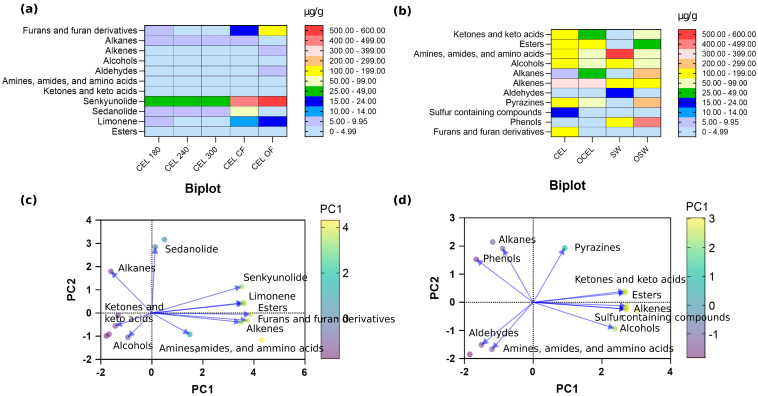
Volatile compounds distribution in alternative curing ingredients. (**a**) Volatile compounds concentration by each chemical group in celery juices. (**b**) Volatile compounds concentration by each chemical group in commercially available alternative curing powders. (**c**) PCA analysis of volatile compounds in organic celery juices. (**d**) PCA analysis of volatile compounds in commercially available alternative curing powder. Abbreviations: CEL = pre-converted conventional celery powder; SW = pre-converted conventional Swiss chard powder; OSW = pre-converted organic Swiss chard powder; OCEL = pre-converted organic celery powder. CEL 180, CEL 240, and CEL 300: organic celery juice produced from celery grown with different levels of fertilizer application (201.74, 268.99, and 336.26 kg/hectare, respectively). CEL CF = non-converted conventional celery juice; CEL OF = non-converted organic celery juice.

**Figure 3:**
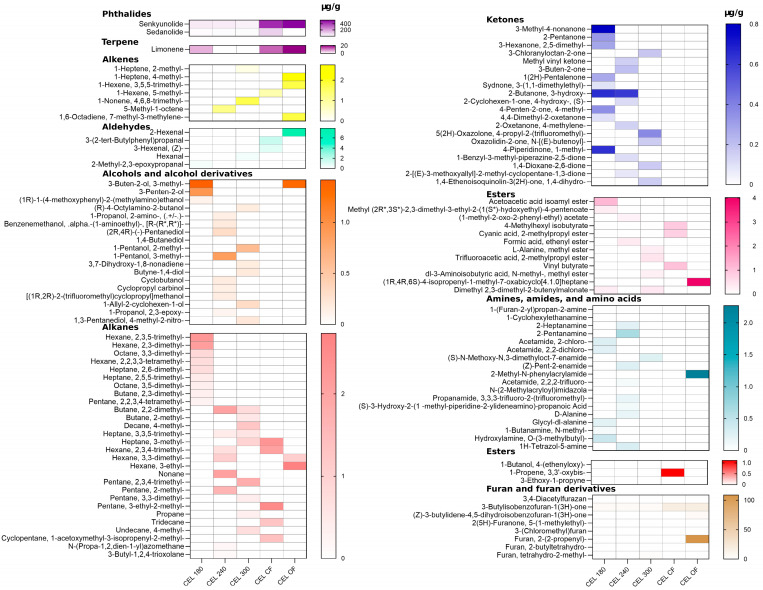
Complete volatile compounds profile in curing powders. Product abbreviations: CEL 180 = organic celery juice with 201.74 kg/hectare (180 lbs./acre) of organic fertilizer treatment, CEL 240 = organic celery juice with 268.99 kg/hectare (240 lbs./acre) of fertilizer treatment, CEL 300 = organic celery juice with 336.26 kg/hectare (300 lbs./acre) of fertilizer treatment. CEL CF = non-converted conventional celery juice; CEL OF = non-converted organic celery juice.

A total of 372 different chemical compounds were observed in the GC-MS total ionizable component chromatograph (Figures S1 and S2). Among these, 153 chemicals were identified using the current NIST library, including 15 ketones, 17 esters, 34 volatile amines and amides, 22 alcohols, 7 alkanes, 30 alkenes, 2 aldehydes, 2 phenols, 2 pyrazines, 2 sulfur-containing compounds, and 2 furanones (Figure 1, Figure 2 and Figure 3). The key and major chemical compositional profiles of VOCs in pre-converted conventional and organic vegetable curing powders are depicted in Figure 2b. A paired PCA analysis of these compounds was conducted as shown in Figure 2d.

In celery powders, pyrazines and furanones were identified as the predominant volatile compounds. In the samples, 2,5-Dimethyl pyrazine had a concentration of 165.91 ± 0.87 µg/g, and 2,6-Dimethyl pyrazine was determined to be 54.65 ± 0.24 µg/g. 3-Butylisobenzofuran-1(3H)-one was detected at a concentration of 196.17 ± 1.01 µg/g in conventional celery powder, but was not identified in the organic celery powder. Interestingly, the organic celery powder contained considerably fewer VOCs than the conventional celery powder, a finding that is in contrast with some previous studies on phytochemicals in celery and organic vegetables [30,31,32].

In pre-converted Swiss chard powders, 2-methoxy-4-vinylphenol (4-vinylguaiacol) was the most abundant aromatic compound identified in both conventional and organic varieties, with concentrations of 139.65 ± 1.76 µg/g and 420.46 ± 2.57 µg/g, respectively (Table 2). Methoxy-4-vinylphenol (4-vinylguaiacol) is a compound that exhibits a sweet, smoky, phenolic, warm, and vanillic aroma [33]. 2,6-Dimethyl pyrazine was identified in OSW but was not present in SW. Overall, the pyrazine content in the organic version of Swiss chard was greater (*p* < 0.05) than in the conventional version, potentially due to more extensive Maillard reactions [20]. This difference may imply potential sensory attribute variations when added to meat products.

### 2.3. Comparison of Non-Converted and Pre-Converted Organic Celery Juices

**Figure 4:**
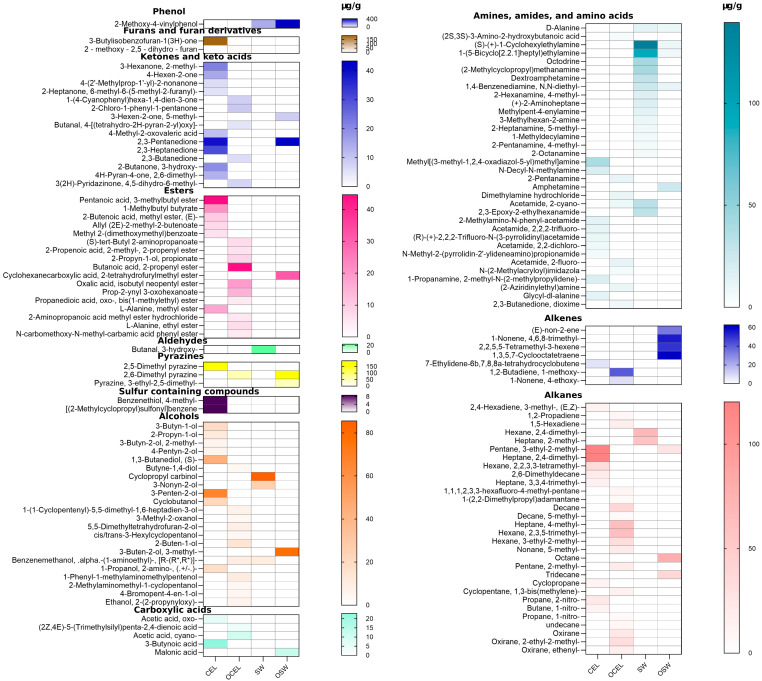
Complete volatile compounds profile in fresh and converted celery juices. Abbreviations: CEL = pre-converted conventional celery powder; SW = pre-converted conventional Swiss chard powder; OSW = pre-converted organic Swiss chard powder; OCEL = pre-converted organic celery powder.

A total of 425 different chemical compounds were detected in the GC-MS total ionizable component chromatograph. Among these, 112 chemicals were identified using the current NIST library, including 2 phthalides; 1 terpene; 7 alkenes; 5 aldehydes; 28 alkanes; 8 furan and furan derivatives; 18 amines, amides, and ammino acids; 20 ketones; and 12 esters (Figure 1, Figure 2, and Figure 4). The key and major chemical compositions of VOCs in pre-converted conventional and organic vegetable curing powders are depicted in Figure 2a. A paired PCA analysis on concentration correlation of these compounds was conducted as shown in Figure 2c.

In pre-converted celery juices, Senkyunolide (structure shown in Figure 1) was present in CEL 180, CEL 240, and CEL 300, with concentrations in descending order of 45.16 ± 0.24, 42.71 ± 0.47, and 30.72 ± 0.58 µg/g, respectively. In non-converted celery juices, including CEL CF and CEL OF, the concentrations were 434.00 ± 2.01 and 518.71 ± 2.4 µg/g, respectively.

Sedanolide (structure shown in Figure 1), another chemical belonging to the group of phthalides, was present in all samples with concentrations of 9.26 ± 0.11, 9.94 ± 0.21, and 7.35 ± 0.17 µg/g in CEL 180, CEL 240, and CEL 300, respectively. The concentration was much higher in CEL CF, at 97.55 ± 0.46 µg/g, but lower in CEL OF, at 2.11 ± 0.01 µg/g (*p* < 0.05).

Limonene (structure shown in Figure 1) is one of the more important terpenes found in celery’s essential oils, which are known to exhibit a fresh, citrusy aroma [28]. In organic pre-converted celery juices, limonene was only present in the CEL 180 sample. In non-converted celery juices, the organic variety contained 21.00 ± 0.23 µg/g of limonene, significantly more (*p* < 0.05) than the conventional variety, which contained 12.87 ± 0.14 µg/g. The identified level was similar as reported in a previous study on terpenes [18].

Other volatile organic compounds (VOCs), such as 3-butylisobenzofuran-1(3H)-one, were present in all celery juices, with concentrations much lower than those in the celery powders (Table 2), possibly due to the concentration effects of drying powders. 2-(2-propenyl)furan, also known as 2-allylfuran, was detected in organic non-converted celery juices at a concentration of 108.92 ± 0.87 µg/g (Table 3). This compound is derived from the thermal degradation of carbohydrates and ascorbic acid, contributing to the caramel-like, sweet, fruity, and nutty notes in various food products [29].

### 2.4. Chemical Composition Differences in Pre-Converted Vegetable Powder and Juices

To investigate patterns and differences in volatile compounds’ compositions in conventional and organic nitrite-source curing ingredients, principal component analysis (PCA) was conducted on both vegetable- and nitrite-source curing powders and pre-converted celery juices (Figure 2). Volatile compounds content in vegetable-source curing ingredients were summed up by conducting PCA analysis. PCA analysis of vegetable curing powders (Figure 2c) revealed that PC1 accounted for 51.14% of the variance and PC2 accounted for 28.32% of the variance. The cumulative proportion of variance explained by PC1 and PC2 was 79.46%, indicating that these two principal components captured the majority of the important information or patterns in the data. In vegetable curing powders, sedanolide was found to be strongly negatively correlated with amines, amides, amino acids, and alcohols. Similarly, senkynolides exhibited a strong negative correlation with ketones and alcohols. These correlations could potentially be explained by the synthesis reactions of phthalides, which involve the amidation of phthalic acids with primary amines and the arylation of aldehydes. [34]. PCA analysis on vegetable curing juices (Figure 2d) revealed that PC1 explained 60.32% and PC2 explained 30.04% of the variance. The cumulative proportion of variance for PC1 and PC2 was 90.36%, indicating that these two components captured most of the important information or patterns in the data, allowing for dimensionality reduction while retaining a significant amount of the original information.

## 3. Discussion

GC-MS/MS coupled with steam distillation and liquid–liquid extraction is a widely adopted method for the extraction of volatile compounds and essential oils from vegetables [35]. The mass identification approach using computational algorithms to identify chromatography peaks and compare spectral data in the NIST library has proven to be an effective tool for analyzing and identifying volatile compounds in large datasets from GC spectrometry. A total of 372 different chemical compounds were observed in the GC-MS total ionizable component chromatograph of pre-converted vegetable-source nitrite curing powder, with pyrazines, furanones, and phenols being the most abundant components. Additionally, 425 different chemical compounds were detected in the GC-MS total ionizable component chromatograph of organic celery juices, with phthalides, terpenes, and furanones being the most abundant components.

As the most predominant compounds identified in pre-converted celery powders, benzofuran derivatives (3-butylisobenzofuran-1(3H)-one) possess a woody, earthy, or slight sweet aroma that represents the smell of alternative celery powder [22]. Furthermore, benzofuran derivatives exhibit a range of biological activities, including antioxidation, antibacterial, antiviral, anti-inflammatory, and anticancer properties [36]. Alkylpyrazines were the second most abundant chemicals identified in celery powders. Alkylpyrazines are considered one of the key compounds formed in Maillard reactions, which contribute to the aroma and sensory attributes of foods with a very low detection threshold of 4 to 490 ppb (*v*/*v*) [20]. 2,5-dimethyl pyrazine in conventional pre-converted celery powder imparts an earthy and roasted aroma, while 2,6-dimethyl pyrazine in organic pre-converted celery powder offers a nutty, coffee-like, and roasted aroma [21].

In pre-converted organic celery juices, phthalides were found to be the most abundant volatile compounds. Senkyunolide contributes to a celery-like aroma that is sweet, earthy, and slightly peppery [24,25]. More recent studies have shown that senkyunolide has potential as a cardio-cerebral vascular drug candidate due to its good heat and acid stability, as well as excellent blood–brain barrier permeability [37,38,39]. This suggests that alternative curing powders containing senkyunolide could offer health benefits when added to processed meat products. Pre-converted celery juice was produced through fermentation. Fermentation reduces the amount of senkyunolide compared to non-fermented celery juices. This reduction could provide insights into the direct and indirect curing processes when celery is used as a meat curing ingredient. Another phthalide, Sedanolide contributes to the characteristic aroma of celery, imparting a sweet, earthy, and slightly peppery scent [26] with an odor threshold of 0.07 ppm [27]. Beyond its unique flavor, sedanolide has been shown to have anti-inflammatory and antitumor properties [40]. It activates the KEAP1-NRF2 pathway, which enhances cellular resistance to oxidative stress and ameliorates hydrogen peroxide-induced apoptotic cell death [41]. Other key aromatic phytochemicals in organic celery juices were studied using PCA analysis. It was found that phenols are strongly negatively correlated with alcohols. This could be explained by their stronger acidity and multiple hydroxy groups, which reduce alcohols during conversion processes [42]. Additionally, pyrazines exhibit a strong negative correlation with amines and aldehydes. This correlation could be due to pyrazine formation from α-amino acids and reducing sugars, which is based on the Maillard reaction and Strecker degradation [43].

Studies have shown that organically grown celery tend to possess greater concentrations of certain bioactive compounds, such as antioxidants, compared to conventionally grown celery, while there were no significant differences in the overall nutritional content, including vitamins and minerals, between organic and conventional celery [44,45]. However, in this study, we found that organic celery powder contained considerably fewer VOCs than conventional celery powder. The potential cause of this difference is possibly due to the variance in processing steps and methods of alternative curing ingredients manufacturers who adopt deodorization procedures [46]. The deodorization process reduces aroma volatiles and can play a vital role in altering sensory scores by reducing volatilized aromatic compounds [47].

Terpenes are one of the most predominant groups of volatile compounds found in fresh, non-converted celery and may impact the flavor of converted celery curing ingredients, as well as meat products cured by those ingredients. Previous research conducted by Sun et al. found that terpenes and aldehydes are the primary contributors to the aroma of celery. The composition of these compounds was correlated with the color and variety of celery, with green celery exhibiting higher concentrations of VOCs. Furthermore, their study revealed that the leaf portions of celery exhibited greater concentrations of VOCs compared to the petioles [18]. Limonene is relatively stable under heat and acidic conditions, except when exposed to high temperatures (approximately 300 °C), as it can isomerize to form α-terpinene [48]. Limonene also has a relatively low sensory threshold, averaging 0.21 ppm, as reported in a study conducted by Ahmed et al. on the flavor threshold of d-limonene in water [49]. Thus, the presence of limonene in celery juices could influence the flavor of processed meat when used as a meat-curing agent. Limonene has been reported to possess health benefits, such as anti-inflammatory effects, by modulating the production of cytokine signaling pathways linked to several diseases [50]. It inhibits the phosphorylation of p38 MAPK, which mediates hydrogen peroxide-induced apoptosis. This action protects lens epithelial cells from oxidative stress through antioxidant and anti-apoptotic pathways [51].

## 4. Materials and Methods

### 4.1. Plant-Source Meat-Curing Materials

Four commercially available conventional and organic pre-converted alternative curing powder were selected in this study, including pre-converted conventional celery powder (CEL; Veg Stable 506, Florida Food Products, FL, USA), organic celery powder (OCEL; 35001, Dianna Foods, Rennes, France), Swiss chard power (SW; Veg Stable 531, Florida Food Products, FL, USA), and organic Swiss chard powder (OSW; Veg Stable 532, Florida Food Products, FL, USA). These pre-converted alternative curing powders are among the most common commercially available products to meat processors in the United States market. Furthermore, three fertilizer treatment levels were used to grow celery to produce organic celery juice, processed by the University of Wisconsin Plant Pathology West Agriculture Experimental Station, located in Madison, WI. Organic celery juice was subsequently converted by the Kerry Group’s pilot plant (Rochester, MN, USA) to pre-convert organic celery juice. Fresh non-converted conventional and organic celery juice were acquired from a local commercial market in Madison, WI. All plant-source meat-curing materials were frozen in a −20 °C freezer before further analysis.

### 4.2. Extraction of VOCs 

Volatile compounds (VOCs) analysis was conducted according to a method described by Wettasinghe et al., with proper modifications accordingly based on sample size [52]. Steam distillation was the method used as it generally extracts more VOCs than solid-phase microextraction methods, as reported in multiple studies [35,53,54,55]. Samples (10 g) were mixed with 10 g of sodium chloride in a 200 mL volumetric tube specifically designed to fit in a fast steam distillation system (SCP DigiPREP Distillation System, SCP Science, Baie-d’urfe, QC, Canada). The distillation was conducted at 60% strength for 300 secs. Distillate (100 mL) was mixed with 150 mL of methylene chloride in a 500 mL separatory flask and mixed vigorously and allowed to stay in ambient temperature (23° C) for 2 h. Internal standards were introduced at 10 µL of 2-octanol and 4a(2H)-Naphthalenol. The methylene chloride layer was vaporized under vacuum using a rotary evaporator (Rotavapor, Buchi, Flawil, Switzerland) at 39.6 °C. When the contents reached a volume of approximately 5 mL, they were removed from the rotary evaporator carefully and mixed with 5 g of ammonium formate to remove any water content in the solution. The concentrate was subsequently transferred into a dark glass vial and stored at −80 °C until analysis.

### 4.3. Detection of VOCs Using a GC-MS/MS System

Gas chromatography–tandem mass spectrometry (GC-MS/MS) analysis was conducted using a GC-MS/MS system coupled with an autosampler (Shimadzu GCMS-TQ 8040NX with AOC-20 plus autosampler Shimadzu Inc., Nakagyo-ku, Kyoto, Japan) supplied with helium gas with a smart switch that uses nitrogen gas in savor mode. Separation of VOCs was conducted on a general-purpose, fused-silica, low-polarity, crosslinked diphenyl dimethyl polysiloxane phase column (Shimadzu SH-I-5MS Capillary Column, 30 m × 0.25 mm × 0.25 um, Shimadzu Inc., Nakagyo-ku, Kyoto, Japan) for semi-volatiles, phenols, amines, residual solvents, drugs of abuse, pesticides, and PCB congeners with operation temperature range (−60 to 330/350 °C). The oven temperature was programed from 45 °C to 240 °C at a rate of 5 °C/min, with an initial and final hold time of 5 min and 10 min, respectively. The total running time was 60 min. For the mass spectrometry detector, the electron ionization energy was set at 70 eV. Mass range, electron multiplier voltage, and scan rate were set at *m*/*z* 33–330, 2000 v, and 20,000 u/s, respectively. Ionization source temperature was maintained at 230 °C [56].

### 4.4. Qualitative and Quantitative Analysis of VOCs

VOCs were identified by matching mass spectral data of sample compounds with an Electron Spray (EI) NIST database (NIST 23 Tandem Mass Spectral Libraries). The area under the curve (AUC) was integrated using Savitzky–Golay methods with the width set at 0.04 min. Each integrated area was compared with the EI database based on spectrum similarity, then manually analyzed based on fragmentation patterns. The results of each alternative cure ingredient sample treatment (TRT) were integrated for comparison using Python (Python version 3.12.7, The Python Software Foundation (Wilmington, DE, USA)) on Spyder (The Scientific Python Development Environment, version 6.0.1, Spyder-IDE.org (accessed on 20 November 2024)) as an Integrated Development Environment (IDE).

### 4.5. Residual Nitrite and Nitrate Measurements

Residual nitrite (NO_2_^−^) and nitrate (NO_3_^−^) were analyzed using high-performance liquid chromatography (HPLC) equipment (ENO-20 NOx Analyzer, Eicom Inc., Kyoto, Japan) coupled with a temperature-controlled autosampler (AS-700, Amuza Inc., San Diego, CA, USA) according to the method described by De González et al. [57], with modifications based on sample size and weight. The HPLC analysis for nitrite and nitrate (NO_x_^−^) was designed based on the Griess nitrite test adopted by the Association of Official Analytical Chemists (AOAC) [57]. Absorption was measured at 540 nm by the UV-Vis detector preinstalled in the HPLC equipment. The sample (5 g) was weighed into 45 mL of pH 7.4 phosphate-buffered saline (PBS) and then split into two equal volume of slurries and centrifuged at 3500× *g* at 4 °C for 5 min (J6-MI centrifuge equipped with JA-25.50 rotor; Beckman Coulter, Indianapolis, IN, USA). After centrifugation, 500 μL of supernatant from each slurry and 500 μL of 100% methanol were mixed, transferred to a 1.5 mL snap cap centrifuge tube, and vortexed for 10 s at 3000 revolutions per minute (rpm) with a digital vortex mixer (cat. no. 0215370, Fisher Scientific, Hanover Park, IL, USA). The samples were then centrifuged for 16 min at 15,000× *g* at 4 °C (Eppendorf 5424 centrifuge, Brinkmann Instruments, Westburg, NY, USA). Supernatants (200 μL) were pipetted into 96-well plates for quantification with the HPLC equipment described above. Quantitative data (area under the curve) were analyzed with PowerChrom (version 16.0, New South Wales, Australia). HPLC carrier pump speed was set at 40 mL/hour and reactor pump speed was set at 13.2 mL/hour. A calibration curve was created using 2, 4, 8, and 16 ppm of sodium nitrite and sodium nitrate. A sodium nitrite standard (8 ppm) was tested at the beginning and end of each run.

### 4.6. pH Measurement

pH measurements were conducted according to a method with modification on sample size [58]. Vegetable powder samples (5 g) were dissolved and mixed with ultrapure water (resistivity of 18.2 MΩ.cm) at a 1:9 ratio using a vortex mixer (Fisherbrand™ Touch Mixer Model 232, Pittsburgh, PA, USA) at 2000 RPM for 30 s. The mixture was then filtered through Whatman #1 filter paper and measured with a pH meter (Fisherbrand™ Accumet™ model 13-620-AE6; Fisher Scientific, Waltham, MA, USA). Calibration of the pH meter was performed using NIST-certified potassium biphthalate buffer (pH = 4.00) and potassium monobasic and sodium hydroxide buffer (pH = 7.0).

### 4.7. Data Analysis

Significant differences among the volatile compounds were analyzed by one-way analysis of variance (ANOVA) using Duncan’s multiple range test (*p* < 0.05). PCA dimensionally reduced all variables into two principal components, PC1 and PC2, to describe data relationships based on eigenvalues (from parallel analysis). A scree plot was employed to validate the principal component analysis (PCA) by determining the proper principal components to retain. All analyses were conducted using R (R version 4.3.3; R Core Team 2024).

## 5. Conclusions

A total of 153 volatile compounds were identified in commercially available curing powders, and 112 volatile compounds were identified in pre-converted organic celery juices. The results of GC-MS/MS and HPLC analyses indicated that vegetable powders could be promising candidates to replace sodium nitrite, offering unique sensory attributes from phytochemicals such as phthalides, phenols, limonene, and benzofurans. Moreover, organic vegetable powders exhibited a distinct profile of aromatic compounds compared to conventional vegetable powders. Sensory studies on alternatively cured meats have primarily focused on sensory panel evaluations. The VOCs in cured meats are highly complex and challenging to pinpoint as contributors to non-meat aftertaste. This study provides practical insights into the specific VOCs that should be examined when conducting VOC analysis and sensory evaluations of processed and cured meats. Pre-converted vegetable juices and powders can both serve as good sources of nitrite for curing meats, but the VOCs profiles in these curing powders are distinctively different. The VOCs profiles in pre-converted vegetable powders may vary based on the manufacturing process, irrespective of conventional or organic growing practices.

Future research on VOCs and sensory evaluation of processed and cured meat products is warranted. This study provides comprehensive insights into the volatile and aromatic compound profiles of currently available conventional and organic pre-converted vegetable powders, as well as organic celery juices. These findings provide substantial reference value for future sensory and quality studies of processed meats. They enhance our comprehension of the complex flavor profiles and contribute to the development of organic processed meat products by offering a detailed understanding of the volatile profiles of these vegetable sources. This study offers potential insights for organic vegetable producers, food ingredient processors, meat processors, and consumers of organic and natural products. It underscores the potential of organic vegetable-derived curing agents to influence the sensory attributes of meat products, while also implicating phytochemicals carried in alternative curing powder that could offer health benefits. This research contributes not only to the development of natural curing agents but also promotes the broader adoption of organic practices within the food industry.

## Figures and Tables

**Table 1:** Physicochemical properties of commercially available alternative curing ingredients. Total ionizable compounds (TIC) chromatography of volatile compounds in alternative curing powders are shown in Figures S1 and S2.

Product ^1,2^	Appearance	Ingredient	pH	Nitrite Content on Label ^3^	Measured Nitrite Content ^3,4^
CEL	Tan to brown free-flowing powder	Cultured celery powder, sea salt	7.88	22,500	22,487
SW	Tan to brown free-flowing powder	Cultured Swiss chard powder, sea salt	8.63	22,500	24,531
OSW	Tan to brown free-flowing powder	Organic Swiss chard powder, sea salt	8.89	22,500	27,711
OCEL	Yellow-brown flowing powder	Sea salt, cultured organic celery juice powder	7.49	20,250–24,750	17,262

^1^ Product abbreviations: CEL = pre-converted conventional celery powder; SW = pre-converted conventional Swiss chard powder; OSW = pre-converted organic Swiss chard powder; OCEL = pre-converted organic celery powder. ^2^ Product suppliers: CEL, SW, and OSW from Florida Foods Inc., and OCEL from Diana Foods Inc. ^3^ Concentration measured as ppm sodium nitrite equivalent. ^4^ Nitrate was not detected.

**Table 2:** Generality compounds table of volatile compounds (VOC) in commercially available alternative curing powder. Approximate quantities of VOCs identified using GC-MS/MS with steam distillation. The Chemical Abstracts Service (CAS) numbers were the compound IDs used to identify the VOCs, which were obtained on the website (https://pubchem.ncbi.nlm.nih.gov/, accessed on 16 November 2024).

Compounds	Commercially Available Alternative Curing Powder ^1^				CAS #	Notes Description
	CEL	OCEL	SW	OSW		
2,5-Dimethyl pyrazine	165.91 ± 0.87	-	-	-	123-32-0	earthy and roasted [20]
2,6-Dimethyl pyrazine	-	54.65 ± 0.24	-	191.41 ± 1.14	108-50-9	nutty, coffee-like, and roasted [20,21]
3-Butylisobenzofuran-1(3H)-one	196.17 ± 1.01	-	-		6066-49-5	woody, spicy, and slightly floral scent [22]
2-methoxy-2,5-dihydro-furan	-	4.93 ± 0.02	-	-	332-77-4	slightly fruity [22]
2-Methoxy-4-vinylphenol	-	-	139.65 ± 1.76	420.46 ± 2.57	7786-61-0	apple, spicy, peanut, wine-like aroma [23]

^1^ Product abbreviations: CEL = pre-converted conventional celery powder; SW = pre-converted conventional Swiss chard powder; OSW = pre-converted organic Swiss chard powder; OCEL = pre-converted organic celery powder. Dash (-) represents not detectable. Values in the table are presented in the form of mean ± standard deviation.

**Table 3:** Generality compounds table of volatile compounds (VOCs) in fresh and pre-converted alternative curing juices quantities (µg/g) of VOCs identified using GC-MS/MS with steam distillation. The Chemical Abstracts Service (CAS) numbers were the compound IDs used to identify the VOCs, which were obtained on the website (https://pubchem.ncbi.nlm.nih.gov/, accessed on 16 November 2024). Total ionizable compounds (TIC) flow diagram of volatile compounds in alternative curing powder are shown in Figure S4.

Compounds	Organic Pre-Converted Celery Juice ^1^			Non-Converted Celery Juice ^2^		CAS #	Notes Description
	CEL 180	CEL 240	CEL 300	CEL CF	CEL OF		
Senkyunolide	45.16 ± 0.24 ^a^	42.71 ± 0.47 ^a^	30.72 ± 0.58 ^b^	434.00 ± 2.01 ^a^	518.71 ± 2.41 ^a^	94596-27-7	celery-like aroma [24,25]
Sedanolide	9.26 ± 0.11 ^a^	9.94 ± 0.21 ^a^	7.35 ± 0.17 ^b^	97.55 ± 0.46 ^a^	2.11 ± 0.01 ^b^	6415-59-4	fresh, green, and slightly spicy aroma of celery [26,27]
Limonene	6.55 ± 0.01 ^a^	-	-	12.87 ± 0.14 ^b^	21.00 ± 0.23 ^a^	138-86-3	fresh, citrusy aroma [28]
3-Butylisobenzofuran-1(3H)-one	4.70 ± 0.47 ^a^	4.52 ± 0.11 ^a^	3.27 ± 0.03 ^b^	17.87 ± 0.25 ^a^	18.21 ± 0.17 ^a^	6066-49-5	woody, spicy, and slightly floral scent [22]
Furan, 2-(2-propenyl)-	-	-	-	-	108.92 ± 0.87	75135-41-0	sweet, green, and slightly fruity aroma [29].

^1^ Abbreviation: CEL 180 = organic celery juice with 201.74 kg/hectare (180 lbs./acre) of organic fertilizer treatment, CEL 240 = organic celery juice with 268.99 kg/hectare (240 lbs./acre) of fertilizer treatment, CEL 300 = organic celery juice with 336.26 kg/hectare (300 lbs./acre) of fertilizer treatment. All celery was harvested in fall 2022, and pre-converted in spring 2023 by Kerry Ingredients in Rochester, MN, USA. ^2^ Abbreviations: CEL CF = non-converted conventional celery juice; CEL OF = non-converted organic celery juice. Dash (-) represents not detectable. Means with unlike lowercase letters (a, b) indicate significant differences (*p* < 0.05) using one-way ANOVA. Values in the table are presented in the form of mean ± standard deviation.

## Data Availability

The data presented in this study are available on request from the corresponding author.

## Supplementary Materials

The following supporting information can be downloaded at: https://www.mdpi.com/article/10.3390/molecules30040835/s1. Figure S1. Chromatography of Volatile compounds in commercially available conventional produced celery powder (labelled in black color) vs. organic produced celery powder (labelled in purple color). Figure S2. Chromatography of Volatile compounds in commercially available pre converted conventional produced Swiss chard powder (labelled in black color) vs. organic produced Swiss chard powder (labelled in purple color). Figure S3. Chromatography of Volatile compounds in commercially available conventional produced cherry powder (labelled in black color) vs. organic produced cherry powder (labelled in purple color). Figure S4. Chromatography of Volatile compounds in organic produced pre-converted celery juice using different levels of fertilizer at 180, 240, and 300 lbs. (labelled in blue, pink, and black color respectively).

## Abbreviations

The following abbreviations are used in this manuscript:

USDA	U.S. Department of Agriculture
HPLC	High-pressure liquid chromatography
GC	Gas chromatography
VOC	Volatile compounds
MS/MS	Tandem mass spectrometry
PCA	Principal component analysis

## References

[1] Keenan D.F., Caballero B., Finglas P.M., Toldrá F. (2016). Pork Meat Quality, Production and Processing on. Encyclopedia of Food and Health.

[2] Karwowska M., Kononiuk A., Wójciak K.M. (2019). Impact of sodium nitrite reduction on lipid oxidation and antioxidant properties of cooked meat products. Antioxidants.

[3] Froehlich D.A., Gullett E., Usborne W. (1983). Effect of nitrite and salt on the color, flavor and overall acceptability of ham. J. Food Sci..

[4] Pierson M.D., Smoot L.A., Robach M.C. (1983). Nitrite, nitrite alternatives, and the control of *Clostridium Botulinum* in cured meats. CRC Crit. Rev. Food Sci. Nutr..

[5] (2024). USDA, 7 CFR 205.606. In U.S.A. https://www.ecfr.gov/current/title-7/section-205.606.

[6] Organic Trade Association (2024). RE: Celery Powder—Handling Subcommittee 2026 Sunset Reviews. https://ota.com/sites/default/files/indexed_files/OTA_HS_CeleryPowder_NOSBSpring2024_AMS-NOP-23-0075_FINAL.pdf.

[7] Santamaria P., Elia A., Serio F., Gonnella M., Parente A. (1999). Comparison between nitrate and ammonium nutrition in fennel, celery, and swiss chard. J. Plant Nutr..

[8] Ballmer-Weber B., Hoffmann A., Wüthrich B., Lüttkopf D., Pompei C., Wangorsch A., Kästner M., Vieths S. (2002). Influence of food processing on the allergenicity of celery: DBPCFC with celery spice and cooked celery in patients with celery allergy. Allergy.

[9] (1979). USDA, 9 CFR 317.17. In U.S.A. https://www.ecfr.gov/current/title-9/section-317.17.

[10] Grand View Research (2023). Organic Meat Market Size, Share & Trends Analysis Report by Meat Type (Poultry, Pork), by Product (Chilled, Preserved), by Distribution Channel (Natural Food Stores, Retailers, Hypermarkets), by Region, and Segment Forecasts, 2023 to 2030.

[11] Mordor Intelligence (2024). Organic Meat Market Size & Share Analysis-Growth Trends & Forecasts (2024–2029).

[12] NOSB (2023). National Organic Standards Board Meeting.

[13] Matallana Gonzalez M., Martinez-Tome M., Torija Isasa M. (2010). Nitrate and nitrite content in organically cultivated vegetables. Food Addit. Contam. Part B.

[14] Ibrahim M.H., Jaafar H.Z.E., Karimi E., Ghasemzadeh A. (2013). Impact of organic and inorganic fertilizers application on the phytochemical and antioxidant activity of Kacip Fatimah (*Labisia pumila* Benth). Molecules.

[15] Klimankova E., Holadová K., Hajšlová J., Čajka T., Poustka J., Koudela M. (2008). Aroma Profiles of Five Basil (*Ocimum basilicum* L.) cultivars grown under conventional and organic conditions. Food Chem..

[16] Ruan S., Luo H., Wu F., He L., Lai R., Tang X. (2023). Organic cultivation induced regulation in yield formation, grain quality attributes, and volatile organic compounds of fragrant rice. Food Chem..

[17] Dong T., Chen X., Wang M., Huang Y., Yi G. (2014). Comparison of volatile aroma compounds in Dwarf Cavendish banana (Musa spp. AAA) grown under organic or traditional cultivation. J. Hortic. Sci. Biotechnol..

[18] Sun Y., Li M., Li X., Du J., Li W., Lin Y., Zhang Y., Wang Y., He W., Chen Q. (2023). Characterization of Volatile Organic Compounds in Five Celery (*Apium graveolens* L.) Cultivars with Different Petiole Colors by HS-SPME-GC-MS. Int. J. Mol. Sci..

[19] Glass C., Silverstein J. (1998). Denitrification kinetics of high nitrate concentration water: pH effect on inhibition and nitrite accumulation. Water Res..

[20] Fors S.M., Olofsson B.K. (1985). Alkylpyrazines, volatiles formed in the Maillard reaction. I. Determination of odour detection thresholds and odour intensity functions by dynamic olfactometry. Chem. Senses.

[21] Hou L., Zhang Y., Wang X. (2019). Characterization of the volatile compounds and taste attributes of sesame pastes processed at different temperatures. J. Oleo Sci..

[22] Miao Y.-h., Hu Y.-h., Yang J., Liu T., Sun J., Wang X.-J. (2019). Natural source, bioactivity and synthesis of benzofuran derivatives. RSC Adv..

[23] Sunao M., Ito T., Hiroshima K., Sato M., Uehara T., Ohno T., Watanabe S., Takahashi H., Hashizume K. (2016). Analysis of volatile phenolic compounds responsible for 4-vinylguaiacol-like odor characteristics of sake. Food Sci. Technol. Res..

[24] Wang K., Liu X., Cai G., Gong J., Guo Y., Gao W. (2023). Chemical Composition Analysis of *Angelica Sinensis (Oliv.) Diels* and its four processed products by ultra-high-performance liquid chromatography coupled with quadrupole-orbitrap mass spectrometry combining with nontargeted metabolomics. J. Sep. Sci..

[25] Zhang J., Cheng M., Xue Y., Lin L., Wang Y., Li B. (2023). Volatile flavour identification and odour complexity of Radix Angelicae Sinensis by electronic nose, integrated gas chromatography–mass spectrometry/olfactometry and comprehensive two-dimensional gas chromatography-time-of-flight-mass spectrometry. Phytochem. Anal..

[26] Kurobayashi Y., Kouno E., Fujita A., Morimitsu Y., Kubota K. (2006). Potent odorants characterize the aroma quality of leaves and stalks in raw and boiled celery. Biosci. Biotechnol. Biochem..

[27] Oguro D., Watanabe H. (2011). Asymmetric synthesis and sensory evaluation of sedanenolide. Biosci. Biotechnol. Biochem..

[28] Jongedijk E., Cankar K., Buchhaupt M., Schrader J., Bouwmeester H., Beekwilder J. (2016). Biotechnological production of limonene in microorganisms. Appl. Microbiol. Biotechnol..

[29] Wailzer B., Kocker J., Wolschann P., Buchbauer G. (2016). Structural features for furan-derived fruity and meaty aroma impressions. Nat. Prod. Commun..

[30] Young J.E., Zhao X., Carey E.E., Welti R., Yang S.S., Wang W. (2005). Phytochemical phenolics in organically grown vegetables. Mol. Nutr. Food Res..

[31] de Oliveira Pereira F., dos Santos Pereira R., de Souza Rosa L., Teodoro A.J. (2016). Organic and conventional vegetables: Comparison of the physical and chemical characteristics and antioxidant activity. Afr. J. Biotechnol..

[32] Ferreira V.B., da Silva T.T.C., Couto S.R.M., Srur A.U.O.S. (2015). Total phenolic compounds and antioxidant activity of organic vegetables consumed in Brazil. Food Nutr. Sci..

[33] Kelly D., Zerihun A. (2015). The effect of phenol composition on the sensory profile of smoke affected wines. Molecules.

[34] Doraghi F., Morshedsolouk M.H., Zahedi N.A., Larijani B., Mahdavi M. (2024). Phthalimides: Developments in synthesis and functionalization. RSC Adv..

[35] Hong Y.S., Son J.H., Jeong J.Y., Song O.Y., Lee J.H., Kim K.S. (2018). Comparison of volatile flavor compounds of *Artemisia annua* L. extracted by simultaneous steam distillation extraction and solid-phase micro extraction. Korean J. Food Preserv..

[36] Abbas A.A., Dawood K.M. (2023). Anticancer therapeutic potential of benzofuran scaffolds. RSC Adv..

[37] Huang Y., Wu Y., Yin H., Du L., Chen C. (2023). Senkyunolide I: A review of its phytochemistry, pharmacology, pharmacokinetics, and drug-likeness. Molecules.

[38] Li Q., Wan J.-B., Zhao L. (2023). Research progress on the pharmacological activities of senkyunolides. Acupunct. Herb. Med..

[39] Qi H., Siu S.O., Chen Y., Han Y., Chu I.K., Tong Y., Lau A.S., Rong J. (2010). Senkyunolides reduce hydrogen peroxide-induced oxidative damage in human liver HepG2 cells via induction of heme oxygenase-1. Chem.-Biol. Interact..

[40] Woods J., Jewell C., O’Brien N. (2001). Sedanolide, A natural phthalide from celery seed oil: Effect on hydrogen peroxide and tert-butyl hydroperoxide-induced toxicity in Hepg2 and Caco-2 human cell lines. Vitr. Mol. Toxicol. A J. Basic Appl. Res..

[41] Hussain M., Sabri R., Zia-Ul-Haq M., Riaz M., Zia-Ul-Haq M., Abdulkreem Al-Huqail A., Riaz M., Farooq Gohar U. (2023). Celery. Essentials of Medicinal and Aromatic Crops.

[42] Xie R., Tu M., Elder T. (2016). Substituent effect of phenolic aldehyde inhibition on alcoholic fermentation by Saccharomyces cerevisiae. Energy Fuels.

[43] Shu C.-K. (1998). Pyrazine formation from amino acids and reducing sugars, a pathway other than Strecker degradation. J. Agric. Food Chem..

[44] Sheng J.-P., Liu C., Shen L. (2009). Comparative study of minerals and some nutrients in organic celery and traditional celery. Spectrosc. Spectr. Anal..

[45] Gąstoł M., Domagała-Świątkiewicz I., Krośniak M. (2011). Organic versus conventional–a comparative study on quality and nutritional value of fruit and vegetable juices. Biol. Agric. Hortic..

[46] Colonia B.S.O., de Melo Pereira G.V., de Carvalho J.C., Karp S.G., Rodrigues C., Soccol V.T., Fanka L.S., Soccol C.R. (2023). Deodorization of algae biomass to overcome off-flavors and odor issues for developing new food products: Innovations, Trends, and Applications. Food Chem. Adv..

[47] Hong J., Kim M.-J., Oh W.Y., Lee J. (2023). Evaluation of deodorization techniques using cyclodextrins on the headspace volatiles and antioxidant properties of onion. Food Chem..

[48] Feng S., Tian Y., Sheng J., Yu J., Lin Y., Hileuskaya K., Kraskouski A., Li H., Lin Z., Shao P. (2024). Enhancing high-temperature stability of limonene-loaded nanostructured lipid carriers with various solid lipids. Food Bioeng..

[49] Ahmed E.M., Dennison R.A., Dougherty R.H., Shaw P.E. (1978). Effect of nonvolatile orange juice components, acid, sugar, and pectin on the flavor threshold of D-limonene in water. J. Agric. Food Chem..

[50] Vieira A.J., Beserra F.P., Souza M.C., Totti B.M., Rozza A.L. (2018). Limonene: Aroma of innovation in health and disease. Chem.-Biol. Interact..

[51] Bai J., Guenther A., Turnipseed A., Duhl T., Yu S., Wang B. (2016). Seasonal variations in whole-ecosystem BVOC emissions from a subtropical bamboo plantation in China. Atmos. Environ..

[52] Wettasinghe M., Vasanthan T., Temelli F., Swallow K. (2001). Volatile flavour composition of cooked by-product blends of chicken, beef and pork: A quantitative GC–MS investigation. Food Res. Int..

[53] Xie Y., He Z., Zhang E., Li H. (2016). Characterization of key volatile odorants in rabbit meat using gas chromatography mass spectrometry with simultaneous distillation extraction. World Rabbit Sci..

[54] Madruga M.S., Elmore J.S., Dodson A.T., Mottram D.S. (2009). Volatile flavour profile of goat meat extracted by three widely used techniques. Food Chem..

[55] Watkins P., Rose G., Warner R., Dunshea F., Pethick D. (2012). A comparison of solid-phase microextraction (SPME) with simultaneous distillation–extraction (SDE) for the analysis of volatile compounds in heated beef and sheep fats. Meat Sci..

[56] Li J., Zhang Q., Peng B., Hu M., Zhong B., Yu C.-w., Tu Z. (2023). Exploration on the quality changes and flavour characteristics of freshwater crayfish (Procambarus clarkia) during steaming and boiling. LWT.

[57] De González M.T.N., Osburn W.N., Hardin M.D., Longnecker M., Garg H.K., Bryan N.S., Keeton J.T. (2012). Survey of Residual Nitrite and Nitrate in Conventional and Organic/Natural/Uncured/Indirectly Cured Meats Available at Retail in the United States. J. Agric. Food Chem..

[58] Korkeala H., Mäki-Petäys O., Alanko T., Sorvettula O. (1986). Determination of pH in meat. Meat Sci..

