# Supplementary material for: Characterization of Volatilized Compounds in Conventional and Organic Vegetable-Source Alternative Meat-Curing Ingredients
Source: Molecules. 2025 Feb 11;30(4):835. doi: 10.3390/molecules30040835 (PMC11858521; doi:10.3390/molecules30040835)
Supplement: Supplementary file 1 [file molecules-30-00835-s001.zip › molecules-3455284-supplementary.pdf]

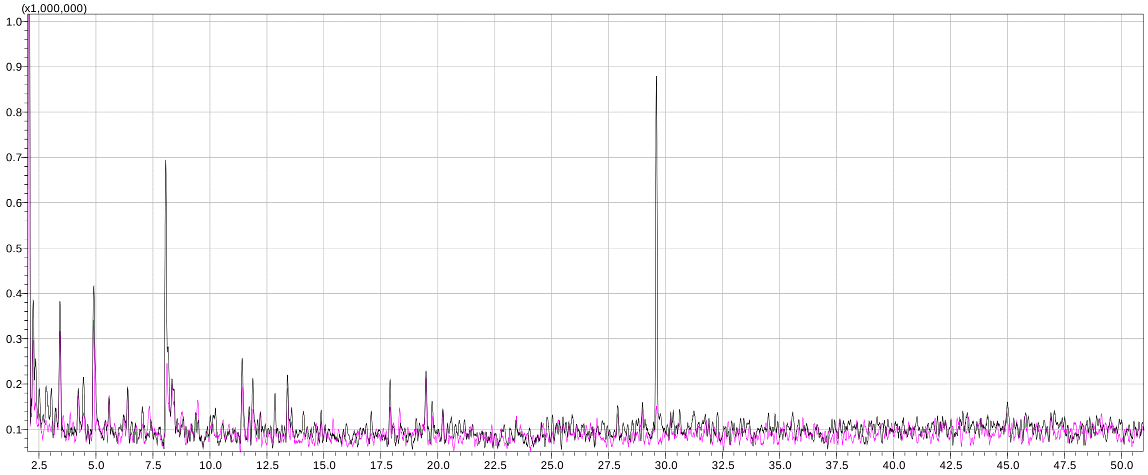

Figure S1. Chromatography of Volatile compounds in commercially available conventional produced celery powder (labelled in black color) vs. organic produced celery powder (labelled in purple color).

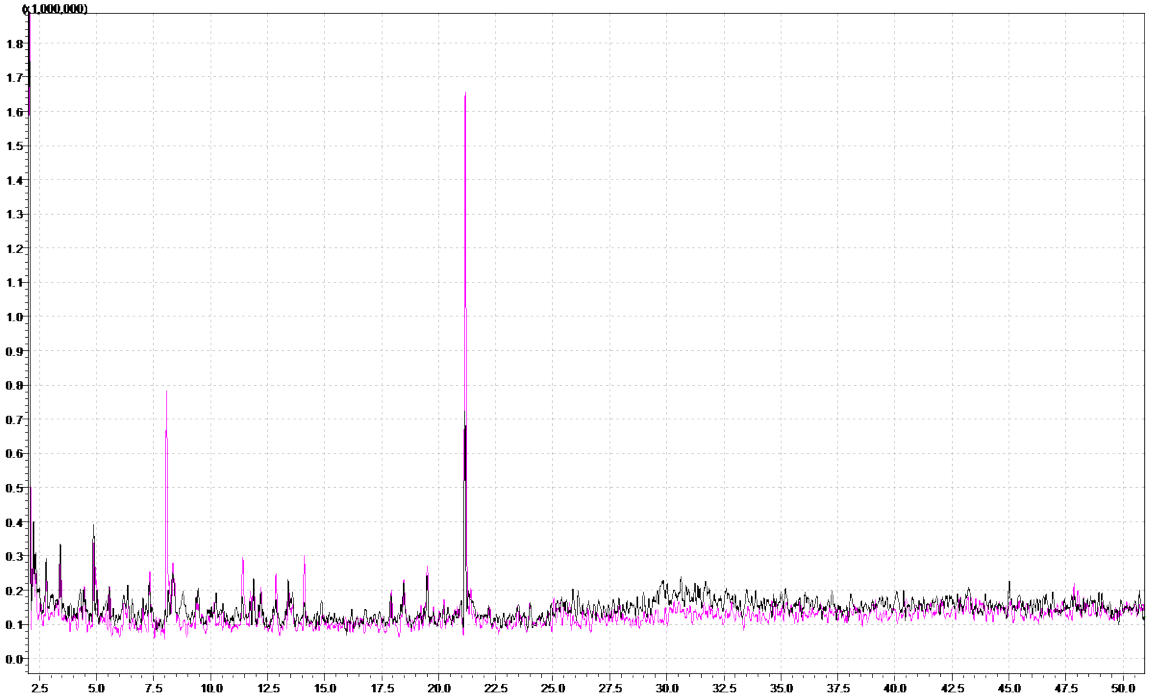

Figure S2. Chromatography of Volatile compounds in commercially available pre converted conventional produced Swiss chard powder (labelled in black color) vs. organic produced Swiss chard powder (labelled in purple color).

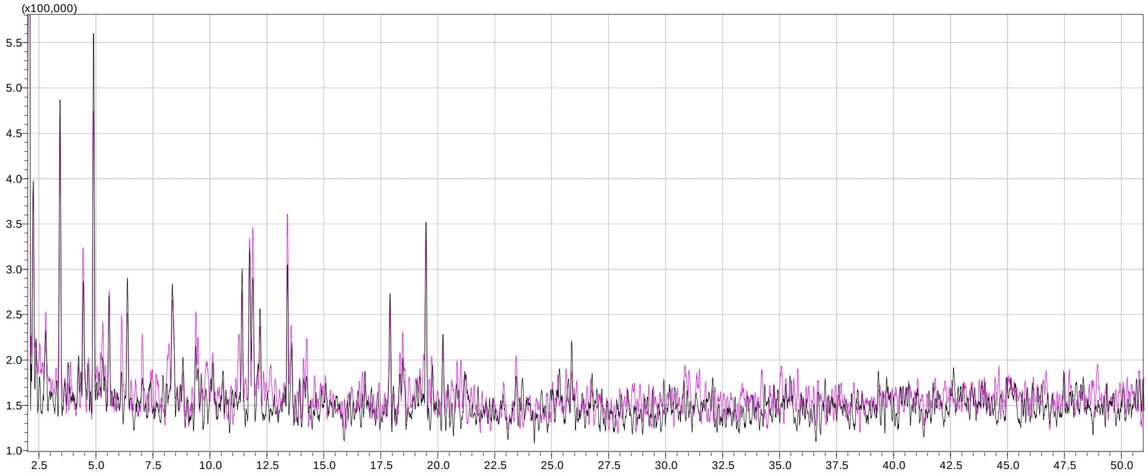

Figure S3. Chromatography of Volatile compounds in commercially available conventional produced cherry powder (labelled in black color) vs. organic produced cherry powder (labelled in purple color).

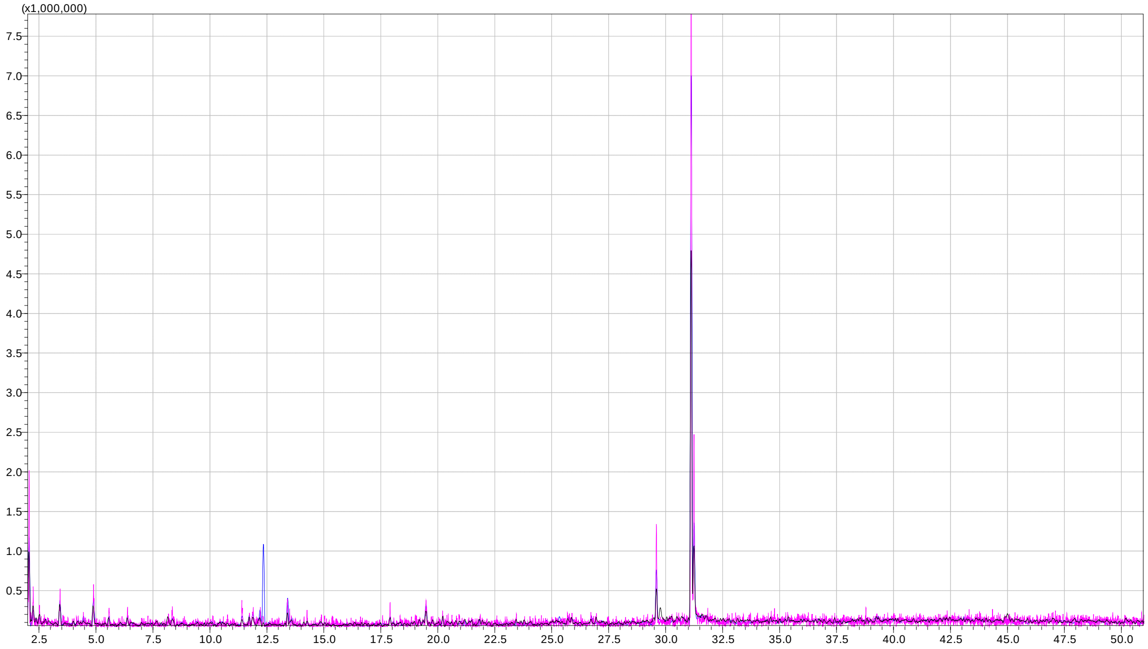

Figure S4. Chromatography of Volatile compounds in organic produced pre-converted celery juice using different levels of fertilizer at 180, 240, and 300 lbs. (labelled in blue, pink, and black color respectively).
